# Applying modern psychometric techniques to melodic discrimination testing: Item response theory, computerised adaptive testing, and automatic item generation

**DOI:** 10.1038/s41598-017-03586-z

**Published:** 2017-06-15

**Authors:** Peter M. C. Harrison, Tom Collins, Daniel Müllensiefen

**Affiliations:** 1 Queen Mary University of London, School of Electronic Engineering and Computer Science, London, E1 4NS United Kingdom; 2 Goldsmiths, University of London, Department of Psychology, London, SE14 6NW United Kingdom; 3 Lehigh University, Department of Psychology, Bethlehem, PA 18015 USA; 4 Music Artificial Intelligence Algorithms, Inc., Davis, CA 95617 USA

## Abstract

Modern psychometric theory provides many useful tools for ability testing, such as item response theory, computerised adaptive testing, and automatic item generation. However, these techniques have yet to be integrated into mainstream psychological practice. This is unfortunate, because modern psychometric techniques can bring many benefits, including sophisticated reliability measures, improved construct validity, avoidance of exposure effects, and improved efficiency. In the present research we therefore use these techniques to develop a new test of a well-studied psychological capacity: melodic discrimination, the ability to detect differences between melodies. We calibrate and validate this test in a series of studies. Studies 1 and 2 respectively calibrate and validate an initial test version, while Studies 3 and 4 calibrate and validate an updated test version incorporating additional easy items. The results support the new test’s viability, with evidence for strong reliability and construct validity. We discuss how these modern psychometric techniques may also be profitably applied to other areas of music psychology and psychological science in general.

## Introduction

Modern psychometric theory provides a remarkable array of tools for ability testing. For example, *item response theory* (IRT) allows scores to be compared between participants who take different tests. *Computerised adaptive testing* (CAT) produces efficient tests that dynamically tailor their difficulty levels to participants of different abilities. *Automatic item generation* (AIG) produces tests with effectively unlimited item banks. Techniques such as these can theoretically produce very powerful and flexible tests of abilities and other latent traits.

Unfortunately, modern psychometric theory has so far only had limited impact on mainstream psychological practice. A number of reasons have been suggested for this, including a lack of mathematically precise thinking in psychology, insufficient mathematical training in psychology education, and an absence of psychological theories sufficiently strong to form the basis of psychometric models^1^. As a result, much psychological research still relies on outdated modes of test construction and validation. At best, this leads to suboptimal testing efficiency; at worst, it leads to incorrect psychological conclusions.

Music psychology is a case in point. There exists a long tradition of musical ability tests, spanning from Seashore’s *Measures of Musical Talents* ^2^ to Kirchberger and Russo’s *Adaptive Music Perception Test* ^3^. However, the vast majority of these tests (e.g. refs 2, 4–9) are built in *classical test theory* ^10^, which psychometricians abandoned long ago in favour of more advanced frameworks such as item response theory.

We propose that psychology, and music psychology in particular, stands to benefit from incorporating more of these psychometric tools. The present paper addresses this possibility. We took a well-established testing paradigm from the music-psychological literature – *melodic discrimination* – and we constructed a new test under this paradigm, making use of a number of modern psychometric techniques including IRT, CAT, and AIG. Together, these psychometric techniques carry substantial potential benefits including flexible test lengths, sophisticated reliability measures, improved testing efficiency, improved construct validity, avoidance of exposure effects, and improved test-construction efficiency. The resulting melodic discrimination test is, to our knowledge, the first musical ability test to incorporate all of these psychometric techniques. It should therefore provide a useful test case for examining how psychological research can benefit from these modern psychometric tools.

## Background

### Melodic discrimination testing

Melodic discrimination is the ability to detect differences between melodies. Many tests have been developed over the years to assess this ability, under names such as *tonal memory tests*, *melodic memory tests*, and *melodic discrimination tests*. However, they all share a common paradigm: participants are played several versions of the same melody, and have to distinguish differences between these versions. The precise task can vary, but most tests use a ‘same-different’ task, where the participant has to decide whether two melody versions are the same or different^4, 6, 11^.

The earliest melodic discrimination tests formed part of *musical aptitude* test batteries, in the tradition of Seashore’s *Measures of Musical Talents* ^2^ (see ref. 12 for a review). The purpose of a musical aptitude test is to assess an individual’s innate capacity for musical success. Musical aptitude tests are still often used as part of entrance exams for school music scholarships, in order to distinguish musical potential from learned ability. Research suggests that musical aptitude scores are indeed good predictors of musical achievement at school^13^ and that it is difficult to change musical aptitude scores through preparation^14^, but the degree to which musical aptitude dictates long-term musical success is still contested^15^.

Melodic discrimination tests are also often used in psychological research. Some of this research uses musical aptitude test batteries^16–18^, whereas other research uses test batteries made expressly for research^19, 20^. Recent examples of such test batteries include the Goldsmiths Musical Sophistication Index (Gold-MSI)^11^, the Montreal Battery of Evaluation of Amusia (MBEA)^21^, the Musical Ear Test (MET)^4^, the Profile of Music Perception Skills (PROMS)^6^, and the Swedish Musical Discrimination Test^5^. By definition, melodic discrimination tests assess the ability to discriminate between melodies, but this ability is often interpreted as reflecting more general cognitive traits, such as perceptual sensitivity to melodies^6^, memory for melodies^11^, and general musical competence^4^.

Melodic discrimination tests therefore have important roles to play both in educational assessment and in psychological research. However, these tests have historically possessed two important limitations: poor construct validity and poor efficiency.

Construct validity concerns how test scores relate to the underlying construct of interest^22^. It is of paramount importance in psychology, as it allows researchers to generalise their results from artificial measures (such as questionnaires or ability tests) to “real” human capacities (such as personality or ability). However, construct validity has received surprisingly little attention in melodic discrimination testing, despite the paradigm’s wide usage. Different studies propose different underlying abilities for these tests, including ‘audiation’^23^, melodic memory^11^, and tonal memory^24^; however, the definitions of these abilities are usually cursory and unsubstantiated. This seriously compromises the construct validity of melodic discrimination testing.

Poor efficiency in melodic discrimination testing has a number of causes. First, in order to achieve sufficient reliability over a wide range of ability levels, the test must contain many items distributed over a wide range of difficulty levels. In traditional fixed-item tests, this means that any one participant will therefore take many items that are either much too easy or too difficult for their ability level, lowering psychometric efficiency^25^. Second, most melodic discrimination tests use multiple-choice questions with only two options, meaning that even minimally able participants can score at least 50%, likewise resulting in lowered psychometric efficiency^26^. Third, melodic discrimination items are slow to administer because of their inherently temporal nature^26^. As a result of this low efficiency, melodic discrimination tests tend to be time-consuming and tiring, diminishing their practical utility.

Fortunately, modern psychometric techniques can make substantial contributions towards both construct validity and test efficiency, the two main limitations of historic melodic discrimination tests. This, coupled with the important role that melodic discrimination testing plays in educational assessment and psychological research, suggests that it may be worthwhile to construct a new melodic discrimination test using these modern psychometric techniques.

### Psychometric theory

#### Item response theory (IRT)

Item response theory (IRT) represents the state of the art in modern test theory (see ref. 25 for a review). Each item is characterised by a finite set of *item parameters*, and each test-taker is characterised by a finite set of *person parameters*. Most common IRT models can be formulated as special cases of the four-parameter logistic (4PL) model, defined as

$$P({X}_{j}=1|\theta ,{a}_{j},{b}_{j},{c}_{j},{d}_{j})={c}_{j}+({d}_{j}-{c}_{j})\frac{\exp \,[{a}_{j}(\theta -{b}_{j})]}{1+\exp \,[{a}_{j}(\theta -{b}_{j})]}$$

where *X*_*j*_ denotes the scored response of the test-taker to item *j* (1 = correct, 0 = incorrect), *θ* is the *ability parameter* for the test-taker, and *a*_*j*_, *b*_*j*_, *c*_*j*_, and *d*_*j*_ are the item parameters for item *j*, respectively termed *discrimination*, *difficulty*, *guessing*, and *inattention parameters* ^27^.

The four item parameters each capture different ways in which items might vary. Items with higher discrimination parameters are better at discriminating between test-takers of different abilities. Items with higher difficulty parameters are harder to answer correctly. The guessing parameter corresponds to the lower performance asymptote (chance success rate), and the inattention parameter corresponds to the upper performance asymptote. Person ability is measured on the same scale as item difficulty, with this scale typically being defined so that the distribution of abilities in the test-taker population has mean 0 and standard deviation 1.

Scoring test-takers’ responses using IRT requires knowledge of the test’s item parameters. This typically requires the IRT model to be calibrated on the basis of response data from a group of test-takers. Once item parameter estimates are obtained, the IRT model can be used to estimate test-taker abilities on a test-independent metric.

In practice, the full flexibility of the 4PL model is not always desirable. Simpler models are typically more efficient to calibrate, requiring less financial investment in the test construction phase. Common simplifications include the three-parameter logistic (3PL) model, where the inattention parameter is constrained to 1, and the Rasch model, where discrimination and inattention parameters are constrained to 1 and guessing parameters are constrained to 0. Whether or not a guessing parameter can plausibly be constrained to 0 depends on the testing paradigm; in particular, multiple-choice paradigms with small numbers of options are very likely to have non-zero chance success rates. In the present work, we constrain the guessing parameter to the reciprocal of the number of response options (*n*), the inattention parameter to 1, and the discrimination parameter to be equal for all items. The resulting model can be expressed as

1 $$P({X}_{j}=1|\theta ,a,{b}_{j},n)=\frac{1}{n}+(1-\frac{1}{n})\frac{\exp \,[a(\theta -{b}_{j})]}{1+\exp \,[a(\theta -{b}_{j})]}$$

where *a* is the shared discrimination parameter. Such models are sometimes termed *constrained* or *modified* 3PL models because they preserve the 3PL model’s non-zero guessing parameter while introducing other constraints on the model parameters (e.g. ref. 28).

IRT brings a couple of important advantages. Unlike classical test theory, IRT allows for the direct comparison of participant scores even when these participants answer different items. This makes it easy for researchers to shorten or lengthen tests without compromising score comparability. It is also a crucial prerequisite for adaptive testing, where participants are administered different items based on their performance levels.

A second advantage of IRT is its sophisticated treatment of *reliability*. Reliability refers to the consistency of a measurement: a reliable instrument is one that delivers consistent results under similar conditions. In classical test theory, reliability is a property of a test with respect to a given test-taker population, and is assessed using measures such as *test-retest reliability* and *Cronbach’s alpha*. These reliability measures have limited generalisability: they are sample dependent, meaning that they cannot be generalised from one test-taker population to another, and they are test dependent, meaning that they cannot be generalised from one test configuration to another. IRT addresses this problem by treating reliability as a function of the items administered and the ability level of the test-taker, with the resulting measure being termed *information*. Information is easy to compute as long as estimates are available for the item parameters. IRT therefore makes it easy to estimate test reliability for new test-taker populations and new test configurations.

IRT-based ability tests are rare in music psychology, but notable exceptions include two melodic discrimination tests^24, 29^, a test of Wagner expertise^30^, a test of notational audiation^31^, and a test of music student competency^32^.

#### Computerised adaptive testing (CAT)

Computerised adaptive testing (CAT) is an approach to ability testing where item selection is algorithmically determined on the basis of the test-taker’s prior responses (see ref. 33 for a review). Item selection typically aims to maximise the information that each item delivers about the test-taker’s true ability.

Several frameworks exist for CAT. The simplest of these, such as the staircase method^34^ and Green’s^35^ adaptive maximum-likelihood procedure, require little pre-calibration but only work for simple tasks, such as psychophysical tests. In contrast, the IRT approach to adaptive testing is flexible enough to be applied to a much wider range of tasks, including melodic discrimination.

Under the IRT framework, adaptive tests typically comprise the following general stages:

1. Make an initial estimate of the test-taker’s ability;
2. Repeat the following steps:
  - Select and administer an item that should deliver maximal information about the participant’s true ability, possibly subject to practical constraints (e.g. not administering the same item twice);
  - Calculate a new estimate of the test-taker’s ability on the basis of their responses;
  - Check whether the stopping criterion is satisfied (e.g. required test length reached); if so, terminate the test.

The primary advantage of CAT is improved testing efficiency. Traditional non-adaptive tests must contain items at a wide range of difficulty levels so as to cater to a wide range of test-taker abilities. This means that any given test-taker will receive some items that deliver low information at their ability level, on account of them being too easy or too hard. In contrast, adaptive tests aim to deliver maximally informative items at each point in the test, resulting in great reliability improvements. As a result, adaptive tests can typically be shortened by 50–80% and still match the reliability of equivalent non-adaptive tests^25, 36^. This effect is particularly pronounced when the test is targeted at a wide range of ability levels, as is common in music psychology.

Several recent musical ability tests incorporate CAT. These include the Adaptive Music Perception Test (AMP)^3^, the Harvard Beat Assessment Test (H-BAT)^37^, and the Battery for the Assessment of Auditory Sensorimotor and Timing Abilities (BAASTA)^38^. However, almost all of these tests use non-IRT procedures which do not generalise well to higher-level cognitive abilities. IRT is the ideal tool for such cases, but we are only aware of one IRT-based CAT in the music-psychological literature: Vispoel’s adaptive tonal memory test^24^. Unfortunately, this test seems to be no longer available.

#### Automatic item generation (AIG)

Automatic item generation (AIG) is an approach to test construction where test items are generated algorithmically together with estimates of their psychometric parameters (see ref. 39 for an overview). It contrasts with traditional approaches to IRT, where items are constructed by hand and psychometric parameters are estimated separately for each item on the basis of empirical response data^25^. To our knowledge, AIG and IRT have yet to be combined in musical ability testing.

One important benefit of AIG is improved efficiency of test construction. Generating items algorithmically avoids the time-consuming process of manual item design. Moreover, predicting psychometric parameters *a priori* means that items do not have to be individually calibrated on human test-takers before use. Both of these characteristics are particularly important for adaptive tests, whose large item banks can otherwise be very expensive to construct and calibrate.

A second important benefit of AIG is improved construct validity. Effective AIG typically relies on identifying the cognitive mechanisms that underlie task performance: this is called *construct representation*, and is an important part of construct validity^40^. This construct representation is used to generate hypotheses about the relationships between structural item features and psychometric item parameters. These hypotheses are then tested on empirical response data. If the hypothesised relationships are supported by the data, this supports the test’s construct representation, and only then can these relationships be used to predict the psychometric characteristics of newly generated items. Construct validity therefore goes hand-in-hand with AIG techniques.

A third benefit of AIG concerns item exposure. Traditional tests have a limited number of items, meaning that participants may become familiar with those items if they take the test multiple times. This can be a problem in psychology research, since the same participant can easily take part in several studies that use the same ability test. However, tests that use AIG can benefit from an effectively unlimited pool of items, making it very unlikely that participants receive the same item again in subsequent test sessions.

This paper takes a *top-down*, *weak theory* approach to AIG. ‘Top-down’ means that item development is driven by an *a priori* theoretical model connecting item features to cognitive processes^41^. ‘Weak theory’ means that item development centres around constructing families of *isomorphic* items: items with differing surface characteristics but similar psychometric characteristics^42^.

Several item response models exist for weak-theory AIG, including the *Identical Siblings Model*, the *Related Siblings Model*, and the *Random-Explanatory Model* ^43^. These models vary in the way that they treat within-family variation in item parameters: the Identical Siblings Model assumes no within-family variation, the Related Siblings Model treats within-family variation as a random effect in a mixed-effects model, and the Random-Explanatory Model treats within-family variation as a combination of fixed and random effects in a mixed-effects model. This paper adopts the Identical Siblings Model for three reasons: (a) its performance closely matches that of its competitors^43^, (b) it is conceptually very simple, and (c) it can be estimated with standard IRT software packages. When combined with the constrained IRT model described in equation (1), the Identical Siblings Model trivially produces the following item response function:

2 $$P({X}_{ij}=1|\theta ,a,{b}_{j},n)=\frac{1}{n}+(1-\frac{1}{n})\frac{\exp \,[a(\theta -{b}_{j})]}{1+\exp \,[a(\theta -{b}_{j})]}$$

where *X*_*ij*_ denotes the test-taker’s scored response to item *i* from item family *j*, *θ* is the test-taker’s ability parameter, *n* is the number of response options, *a* is the shared discrimination parameter across all item families, and *b*_*j*_ is the difficulty parameter for item family *j*. Note that the assumption of zero within-family variation in item parameters means that the expression for the item response function is independent of *i*.

We approach our AIG task as follows. First, we describe the generic form of the items used in our melodic discrimination test. We then outline the cognitive model of melodic discrimination that forms the basis of our AIG system. We use this cognitive model to identify which item features should significantly affect item difficulty (*radicals*) and which should have minimal effect on difficulty (*incidentals*). Radicals and incidentals are then manipulated to define 20 (later 32) item families, constructed so as to cover a wide range of difficulty levels, with item difficulty being hypothesised to be constant within item families but to differ across families. We then develop a protocol for automatically generating melodic discrimination items within these families. Lastly, we calibrate the psychometric parameters of these item families in two empirical studies (Studies 1 and 3), and validate the performance of the resulting adaptive melodic discrimination test in two further empirical studies (Studies 2 and 4).

## Test design

### Generic item form

Most melodic discrimination tests use a ‘same-different’ paradigm, where the participant is played two versions of the same melody and is asked whether they are the same or different^4, 6, 29^. This paradigm is appealing for its simplicity, but is problematic for IRT in that task performance depends both on task ability and on the participant’s individual decision threshold^29^. Other tests use a paradigm where participants are played two melodies that differ by one note, and their task is to identify which note differed^5, 24^. This eliminates the decision threshold problem, but may introduce an unwanted task dependency with numerical fluency.

**Figure 1:**
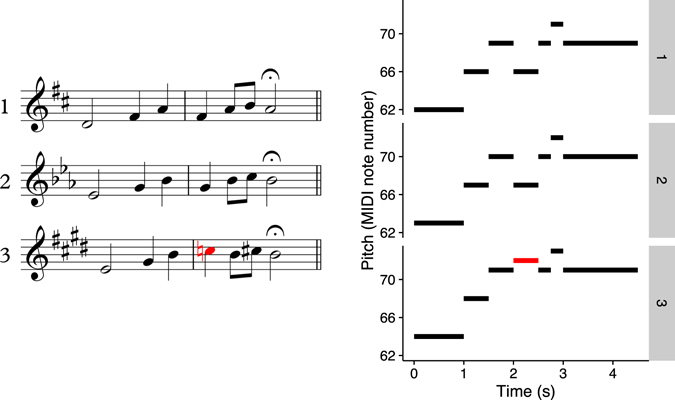
Example 3-AFC melodic discrimination item, in traditional music notation (left) and piano-roll representation (right). The third melody is the odd-one-out, and the altered note is highlighted.

In the present research we therefore introduce a three-alternative forced-choice (3-AFC) melodic discrimination paradigm, which does not require such a decision threshold. In each trial, the participant is presented with three versions of the same melody. Two of these versions possess the same interval structure and are called *lures*, but one version has exactly one note altered, and is called the *odd-one-out*. These three versions can occur in any order, and the participant’s task is to identify which version was the odd-one-out. An example trial is illustrated in Fig. 1.

### Cognitive model

Many previous studies in experimental psychology have used the melodic discrimination paradigm to explore melody perception and cognition^29, 44–48^. These studies can provide a useful cognitive basis for melodic discrimination testing. Here we adopt the cognitive model of melodic discrimination proposed by Harrison and colleagues^29^, which identifies four primary cognitive processes that underlie melodic discrimination: perceptual encoding, memory retention, similarity comparison, and decision-making.

Perceptual encoding occurs first, with the listener forming a cognitive representation of the melody as it is played. This involves extracting a number of different features from the melody, such as pitch content, interval content, melodic contour, and harmonic structure. Next, for all melodies aside from the last melody in the trial, the participant must retain the melodies’ cognitive representations in working memory. Similarity comparisons are then performed between these cognitive representations, making use of the different feature representations that were formed in perceptual encoding and stored in memory retention. In the 3-AFC paradigm, we suggest that each melody version is compared with every other version, producing three similarity comparisons in total. Lastly, the participant performs a decision-making process to determine which melody was the odd-one-out, on the basis of these similarity judgements. Two of these melody pairs must be different, and one must be the same; the participant’s task is therefore to identify the most similar pair, and then the odd-one-out must be the melody not contained within this pair. Other decision strategies are possible, but we suggest that these alternative strategies have similar psychometric implications.

### Implications for item difficulty

Item features that impair any of the four primary cognitive processes in the melodic discrimination task should be expected to increase item difficulty. Particularly important item features are melodic complexity, melodic similarity, conformity to cultural schemata, and pitch transposition^29^. More complex melodies place higher demands on the limited capacity of working memory, and hence result in more difficult items^47^. Increased contour similarity and tonal similarity between melody versions makes the similarity comparison task more demanding, hence increasing item difficulty^49^. Conformity to cultural schemata aids perceptual encoding and memory retention, thereby decreasing item difficulty^47, 48^. Transposition impairs perceptual encoding and similarity comparison, hence increasing item difficulty^48^.

Melodic complexity, similarity, conformity to cultural schemata, and transposition can therefore all be described as radicals: they are features that should contribute to item difficulty. The aim of varying the radicals is to produce a suitable range of item difficulties for the adaptive test. In this research we manipulate the first two radicals (complexity and similarity) while keeping the second two (conformity to cultural schemata and transposition) constant.

Complexity is operationalised as the number of notes in the melody (termed *length*). Longer melodies are more complex, and should therefore result in higher item difficulties. Similarity is operationalised in terms of two dichotomous variables: whether the altered melody differs in pitch contour from the original (*contour violation*) and whether the altered note leaves the home key of the original melody (*tonality violation*). Contour and tonality violations should decrease similarity, hence decreasing item difficulty.

Incidentals are features that are expected not to contribute substantially to item difficulty. Manipulating incidentals introduces variation into test items, hence reducing exposure effects and improving generalisability. The primary incidental manipulated in the present research is the *base melody* used for each item. This ensures that the participant is always discriminating between unfamiliar melodies. We also treat the position of the odd-one-out as an incidental, and randomise it across all trials to prevent it from becoming a response cue.

Prior research usually treats melodic discrimination as a unidimensional ability, occasionally split into (typically correlated) rhythm and pitch subcomponents^4, 23^. To maintain a viable scope for the present paper, we focus on the detection of pitch differences, and leave rhythmic discrimination to future work. Rhythmic discrimination aside, the cognitive model described above still describes four primary cognitive processes behind melodic discrimination, and individual differences in each of these cognitive processes could lead to a multidimensional melodic discrimination ability. For example, an individual might be good at discriminating very similar melodies, but bad at retaining complex melodies. Later in this paper we examine this hypothesis empirically.

### Item families

Version 1 of the adaptive melodic discrimination test comprises 20 item families. Each item family corresponds to a unique combination of the three radicals: two dichotomous radicals (contour violation, tonality violation), and five levels of length (6, 7, 9, 12, and 16 notes). Transposition is kept constant, with the same starting key being used for all items (D major), and each successive melody in the 3-AFC trial being transposed one semitone higher than its predecessor. Conformity to cultural schemata is also kept constant, with the chosen musical idiom being Irish folk melodies.

Version 2 of the adaptive melodic discrimination test expands Version 1 by introducing three more length levels (3, 4, and 5 notes). These new length levels are factorially combined with the remaining radicals, producing 12 new item families and bringing the total number of item families to 32.

### Item generation protocol

The purpose of the automatic item generation protocol is to provide an (effectively) unlimited supply of items for each item family. This protocol comprises three main steps: generating the base melodies, generating altered melodies, and synthesising the corresponding audio.

Base melodies are generated algorithmically by the computational model Racchman-Jun2015 (Random Constrained CHain of MArkovian Nodes)^50, 51^, which takes as input a corpus of source music in a particular musical style, calculates a matrix of transition probabilities between musical events, and uses this transition matrix to generate new musical extracts in the style of the source corpus. The source corpus used here is the collection of Irish folk melodies from the Essen collection^52^ in simple triple time. Two constraints are placed on base melody generation. One constraint is that melodies at a particular length level (e.g. 6 notes) should all occupy the same number of musical beats, hence keeping note density constant. Another constraint is that no more than two consecutive note events should come from the same melody, reducing the probability that the algorithm will replicate a segment of a source melody note for note. A pilot study with 20 participants (mostly university students with limited familiarity with Irish folk music) and 80 trials per participant found no significant difference in perceived stylistic success between melodies generated by the computational model and melody extracts from the source corpus (Welch *t*-test, *t*(74.2) = 0.71, *p* = 0.48). This suggests that the generated melodies should be sufficiently realistic for use in the melodic discrimination test.

Four altered melodies are produced for each base melody, one satisfying each combination of contour violation and tonality violation. Alterations are produced by modifying the relative pitch of exactly one note in the base melody, with the following constraints:

1. For melodies with lengths of 5 notes or fewer, neither the first nor the last note are available for alteration.
2. For melodies with lengths of 6 notes or longer, neither the first two nor the last two notes are available for alteration.
3. The altered note must not differ from the original note by more than 6 semitones.
4. The altered note must be between an eighth note and a dotted half note in length, inclusive.

A search algorithm attempts to find alterations that satisfy these constraints while minimising the displacement distance between the altered note and the original note. If four altered melodies cannot be found for the base melody, the base melody is discarded and the process starts again with a new base melody.

All stimuli are synthesised from MIDI with identical piano timbre and a tempo of 120 beats per minute. The three melodies within each trial are always separated by 1 s of silence, and the first melody within a trial always takes the key of D major. Three audio stimuli can be generated from the same combination of altered melody and base melody, since the odd-one-out can come either first, second, or third in the 3-AFC trial. Example stimuli can be found in the supplementary materials.

## Studies

### Study 1: First calibration

The primary aim of this first study was to estimate psychometric parameters for Version 1 of the adaptive melodic discrimination test. This involved administering automatically generated items to a large number of participants and fitting an IRT model to the resulting response data. This IRT model could then be used to estimate psychometric parameters for new items.

The automatically generated items spanned a wide range of difficulties, and so effective estimation of item parameters required the participants to span a wide range of ability levels. We addressed this by recruiting both adults and schoolchildren. The schoolchildren were expected to be less cognitively developed than the adults, and therefore to have lower melodic discrimination abilities. We assumed that, otherwise, melodic discrimination should be similar in schoolchildren and in adults.

This study also provided an opportunity to investigate the viability of the proposed adaptive melodic discrimination test. The test’s underlying psychometric assumptions were assessed using various tools from the IRT literature, such as unidimensionality tests and model fit statistics. The reliability of item difficulty predictions was assessed through the inspection of item discrimination parameters. Construct validity was assessed in terms of *construct representation* and *nomothetic span*: *construct representation* was investigated by regressing item difficulties on structural item features, while *nomothetic span* was investigated by correlating participant abilities with other participant attributes^40^. Lastly, item difficulties were compared to participant abilities to determine the test’s suitability for different ability levels.

### Method

#### Participants

Two participant groups were used: one group of adults (*N* = 158) and one group of schoolchildren (*N* = 266). The adults were recruited via social media and word-of-mouth, and were rewarded by a prize draw for a £100 (≈$125) gift voucher as well as the chance to see feedback on their melodic discrimination skills. They were approximately evenly split by gender (65 males, 87 females, six anonymous) and ranged in age from 18 to 77 years (*M* = 34.4, *SD* = 14.6). The schoolchildren, meanwhile, participated as part of a wider study investigating a broader range of academic and musical skills^53^. All schoolchildren were female, and ranged in age from 6 to 18 (*M* = 14.5, *SD* = 1.79).

#### Materials

*Melodic discrimination test*: This study used a non-adaptive instance of Version 1 of the melodic discrimination test. Twenty base melodies were generated for each of the five length levels, which were then crossed with contour violation (two levels), tonality violation (two levels), and position of the odd-one-out (three levels) to produce 1,200 items in total.

*Musical training questionnaire:* The musical training questionnaire was sourced from the *Goldsmiths Musical Sophistication Index* (Gold-MSI)^11^ self-report measure. It comprised seven items addressing the participant’s formal musical background as well as their performance ability. Scores on these seven items were aggregated to produce a numeric musical training score for each participant.

#### Procedure

Data were collected using the *Concerto* platform^54^. Adults participated online, agreeing to wear headphones and to take the test in a quiet room free from interruptions. Schoolchildren participated in quiet classrooms wearing standardised headphones (Behringer, HPM1000).

Adult testing sessions lasted approximately 12 minutes each. Sessions began with the melodic discrimination test and concluded with two short questionnaires. The first was the Gold-MSI musical training questionnaire, described above; the second comprised some basic demographic questions (age, gender, occupational status). Upon completion of the questionnaires adult participants were presented with their total melodic discrimination scores.

Schoolchildren testing sessions lasted approximately an hour each, and included the melodic discrimination test alongside a number of other listening tests and questionnaires. The questionnaires included the Gold-MSI musical training questionnaire and basic demographic questions. Results from other listening tests and questionnaires are not reported here. The schoolchildren received no feedback for their scores.

For all participants, the melodic discrimination test began with a training phase, which included instructions, audio examples, and two practice trials. Participants were free to repeat the training phase if they felt unsure about the task procedure. After completing the training phase, all participants answered 20 randomly selected items, with the constraints that each item family was represented exactly once and that no base melody was heard more than once.

#### Ethics

All experimental protocols in this and subsequent studies were approved by the Ethics Committee of Goldsmiths, University of London, and all experiments were performed in accordance with the relevant guidelines and regulations. Informed consent was obtained from all participants prior to participation.

### Results

Response data were modelled using the item response model described in equation (2) with *n* = 3. The model was fit with approximate marginal maximum likelihood, using the *ltm* package^55^ in the statistical software environment *R* ^56^.

Model quality was assessed in several ways. First, model fit for the different item families was assessed using Yen’s^57^ *Q*_1_ statistic with 10 ability groups, taking 500 Monte Carlo samples to estimate the distribution of the statistic under the null hypothesis, and calculating significance levels using Bonferroni correction. No item families exhibited statistically significant levels of poor fit, and this result proved robust to variation of the number of ability groups. Model fit and conditional independence were then assessed by computing the model fit on the two-way and three-way margins, after Bartholomew^58^. Out of the 760 pairs of items and response patterns examined for the two-way margins, only one pair (0.13% of the total) was flagged for poor fit by Bartholomew’s^58^ criterion (test statistic greater than 4.0). For the three-way margins, meanwhile, only 67 out of 9,120 triples (0.73%) were flagged for poor fit. Overall, these results suggested that the model fit well and satisfied the assumption of conditional independence.

Unidimensionality was then tested using *modified parallel analysis* ^59^. This involved calculating the second eigenvalue of the tetrachoric correlation matrix for the response data, and comparing this eigenvalue to a Monte Carlo simulation of its distribution under the null hypothesis. The results showed no evidence for multidimensionality (500 Monte Carlo samples, *p* = 0.49).

Effective AIG using weak theory requires that items in the same family possess similar item difficulties. In the Identical Siblings Model, similar item difficulties lead to higher discrimination parameters^43^. The observed global discrimination parameter for the melodic discrimination test was relatively high (1.31), suggesting similar difficulties within item families and hence suitability for AIG.

**Figure 2:**
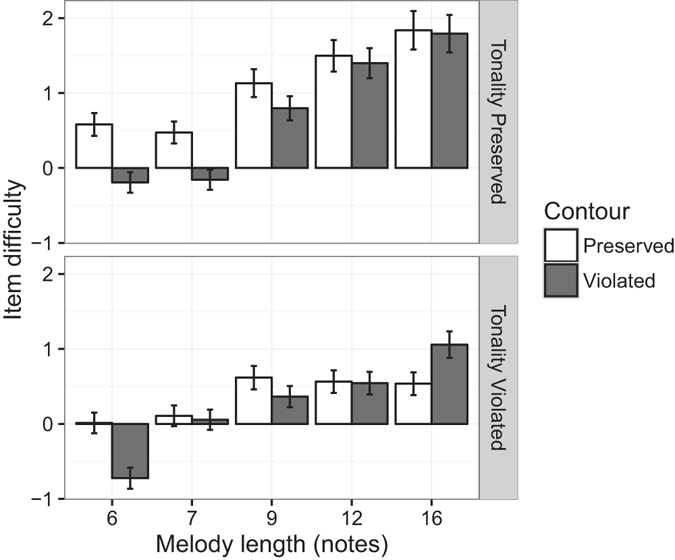
Item difficulty as a function of melody length, contour violation, and tonality violation (test Version 1). Error bars denote one standard error.

A linear regression model was then constructed to investigate the effects of the radicals on item difficulty (Fig. 2). The model predictors comprised the three radicals (melody length, contour violation, and tonality violation), as well as all pairwise interactions between these radicals. Melody length was treated as a continuous variable, and linearly scaled to take a mean of 0 and a standard deviation of 1. The resulting regression model was statistically significant, *F*(6, 13) = 21.99, *p* < 0.001, with an adjusted *R* ^2^ of 0.869 (Table 1). As hypothesised, longer melodies were significantly harder than shorter melodies, while contour and tonality violations significantly reduced item difficulty (tonality violations more so than contour violations). However, the interaction effects show that the impact of contour and tonality violations depended on melody length. As melody length increased, contour violations reduced difficulty less, but tonality violations reduced difficulty more.

**Table 1:** Linear regression model predicting item difficulty from the radicals (test Version 1).

Predictor	β	*SE*	*t*	*p*
(Intercept)	1.104	0.110	9.997	<0.001***
Melody length	0.494	0.098	5.034	<0.001***
Contour violation	−0.377	0.156	−2.411	0.031*
Tonality violation	−0.737	0.156	−4.716	<0.001***
Melody length * contour violation	0.319	0.113	2.814	0.015*
Melody length * tonality violation	−0.282	0.113	−2.489	0.027*
Contour violation * tonality violation	0.269	0.221	1.216	0.246

*Note*. *p < 0.05. ***p < 0.001. Regression coefficients are standardised.

Melodic discrimination ability scores (expected *a posteriori*) were calculated for all participants on the basis of the IRT model. A linear regression was then conducted to investigate how sample group and musical training contributed to task performance. Nine participants were excluded on account of missing data, and musical training scores were linearly scaled to a mean of 0 and a standard deviation of 1. The regression was statistically significant, *F*(3, 411) = 118, *p* < 0.001, and had an adjusted *R* ^2^ of 0.459 (Table 2). This model indicated that musical training and membership of the adult group were both positively associated with task performance, but that musical training was a stronger predictor of performance for the adult group than for the child group.

**Table 2:** Linear regression model predicting melodic discrimination ability (Study 1).

Predictor	β	*SE*	*t*	*p*
(Intercept)	−0.293	0.039	−7.515	<0.001***
Adult (vs. child)	0.710	0.066	10.727	<0.001***
Musical training	0.234	0.043	5.425	<0.001***
Adult * musical training	0.151	0.064	2.369	0.018*

*Note*. *p < 0.05. ***p < 0.001. Regression coefficients are standardised.

**Figure 3:**
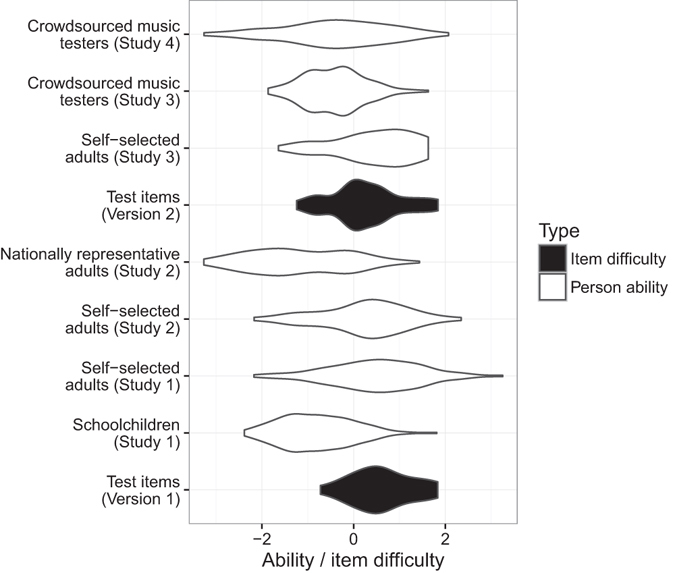
Person ability and item difficulty distributions for the four studies and two test versions. As is conventional, the widths of the ‘violins’ are proportional to the densities of the smoothed distributions.

Ability scores for the two sample groups were then compared with the distribution of item difficulties for test Version 1 (Fig. 3). The distribution of item difficulties matched the adult ability distribution relatively well, but matched the children less well, who performed significantly worse than the adults (mean child ability = −0.364, mean adult ability = 0.614, Welch *t*-test, *t*(273.6) = −13.796, *p* < 0.001).

### Discussion

The purpose of this first study was to estimate psychometric parameters for Version 1 of the adaptive melodic discrimination test. The results suggest that this process was successful. IRT assumptions of model fit, item conditional independence, and unidimensionality were satisfied, and the high item discrimination indicated that item difficulty could be predicted well by item-family membership.

Construct representation is the aspect of construct validity concerning the cognitive mechanisms that underlie task performance^40^. We investigated construct representation by regressing item difficulties on structural item features. The regression model showed that longer melodies produced harder items, and that contour violations and tonality violations produced easier items, as hypothesised. The results support the cognitive model of the melodic discrimination task and hence its construct representation.

The regression model also found that the effect of contour violations decreased for longer melodies, whereas the effect of tonality violations increased for longer melodies. These results were not predicted in test construction, but are consistent with prior research. An interaction between contour violation and melody length has been described by Edworthy^60^, who found that listeners are better at detecting contour violations than interval violations for short melodies, but better at detecting interval violations than contour violations for long melodies. Edworthy^60^ suggested that this is because contour can be encoded independently of tonal context, unlike interval information, whose encoding benefits from the greater tonal context available in longer melodies. Meanwhile, the interaction between tonality violation and melody length is consistent with previous work showing that tonality violations are easier to detect with greater tonal context^61^. As melody length increases, tonal context increases, and hence tonality violations are more salient, as observed. Both of these effects are consistent with our cognitive model.

Nomothetic span is a complementary aspect of construct validity concerning how test-taker scores relate to other variables^40^. We investigated nomothetic span by regressing test scores on other participant attributes. Musical training was positively associated with melodic discrimination ability, consistent with prior research^4, 6, 11^. Membership of the adult group positively predicted melodic discrimination ability, perhaps because adults tend to possess better developed cognitive abilities, but also perhaps because the adults were better motivated (adults opted in for testing, whereas children could only opt out) and less tired (the total length of the testing session was shorter for adults than for children). These results are consistent with our conception of melodic discrimination ability, and hence support the test’s nomothetic span. However, the limited number of comparison variables limits the conclusions that can be drawn; nomothetic span was therefore explored further in Study 2.

It is interesting that musical training was more strongly associated with melodic discrimination performance for adults than for children. One explanation is that the test’s difficulty was better suited to the adults than to the children, resulting in higher discrimination power, higher reliability, and hence higher correlations between test scores and musical training for the adult group.

Our cognitive model describes four primary cognitive processes behind melodic discrimination. Individual differences in each of these cognitive processes could lead to a multidimensional melodic discrimination ability. However, the results showed no evidence for multidimensionality. There are several possible interpretations for this: (a) multiple abilities exist, but they are highly correlated and hence difficult to distinguish psychometrically; (b) multiple abilities exist, but some are more prone to individual differences than others; (c) multiple abilities exist, but not all are tested fully by the melodic discrimination task. For practical purposes, however, it seems that melodic discrimination can be treated as a unidimensional ability.

Comparing the distributions of ability scores and item parameters indicated that the item bank suited the adult sample group well, but did not contain enough easy items for the child group. This limitation was subsequently addressed in Study 3. First, however, a study was conducted to validate this first version of the adaptive melodic discrimination test.

### Study 2: First validation

This purpose of this study was to gather three types of validation information about the adaptive melodic description test. First, we aimed to gather population norms for the test, so that future test-takers could be evaluated with respect to the general population. The second aim was to investigate the test’s nomothetic span, the matter of how test-taker scores relate to other variables. The third aim was to investigate the test’s reliability.

Different participant populations are relevant for different scenarios in psychology research and in educational testing. One important population is that of the country as a whole. Here we estimated norms for this population by testing a nationally representative group sourced by a market research company. However, many music psychology studies do not randomly sample from the entire population, but instead use self-selected sample groups. Self-selected participants are more likely to be actively interested in music, and may correspondingly have better musical listening abilities. We therefore also collected norms for a sample group of self-selected participants recruited by word of mouth and by social media.

Nomothetic span had received preliminary analysis in the previous study, but we explored it further here. Three aspects of nomothetic span were assessed: *concurrent validity*, *convergent validity*, and *divergent validity*. Concurrent validity is demonstrated when test scores correlate well with test scores from a pre-established test of the same ability. Here we investigated concurrent validity with a shortened version of the Musical Ear Test^4^, which has been shown to discriminate reliably between professional musicians, amateur musicians, and non-musicians, as well as predicting various aspects of musical expertise. Convergent validity means that test scores correlate appropriately with measures of other related abilities. We assessed convergent validity by testing how melodic discrimination scores related to musical training; previous research indicated that musical training should be positively associated with melodic discrimination scores^4, 6, 11^. Lastly, divergent validity is shown when test scores show an appropriate lack of correlation with measures of theoretically unrelated abilities. Here we assessed divergent validity using a low-level psychoacoustic task where the participant had to determine the order of short successive tones.

A reliable test is one that delivers similar results when administered under similar situations. In this study we measured test reliability in two ways. First, we used the IRT model to estimate the statistical uncertainty of its ability estimates. Second, we administered the test twice to a subset of participants, and investigated how well test scores correlated between the two administrations (test-retest reliability). We placed a special focus on investigating reliability at different possible test lengths, aiming to see whether the test could still perform well when shortened.

### Method

#### Participants

The study used two sample groups: a nationally representative group and a self-selected group. The nationally representative group was recruited by the market research company ‘Qualtrics’ (www.qualtrics.com) and comprised 185 UK residents who participated online in exchange for a small financial reward. This group was nationally representative in terms of age, gender, occupation, and geographic location. Participant ages ranged from 18 to 73 (*M* = 43.1, *SD* = 13.9), with approximately half (92 out of 185) of the participants being female. No participants reported hearing problems.

The self-selected group numbered 53 individuals recruited by social media and word of mouth. Most (30 out of 53) of this group were students, with slightly more females than males (31 vs. 22). None reported hearing problems. All self-selected participants were rewarded for participation by entry into a raffle for a £100 (≈$125) gift voucher.

#### Materials

*Melodic discrimination test:* This study used an adaptive implementation of Version 1 of the melodic discrimination test, using the item bank calibrated in Study 1. The CAT algorithm worked as follows. First, each participant was administered an item from the item family with the lowest item difficulty. After each item was administered, the participant’s ability was estimated using Bayes modal estimation, using a normally distributed prior with mean 0 and standard deviation 1. Successive items were then selected from the item family whose difficulty level was closest to the present ability estimate. The test terminated after administering 20 items. At this point the participant’s ability was re-estimated using weighted maximum-likelihood estimation^62^, so as to remove the bias of the Bayesian prior. The test was implemented using the R package *catR* ^27^ on the Concerto platform^54^.

*Musical Ear Test:* The Musical Ear Test (MET)^4^ is a musical listening test that uses the ‘same-different’ discrimination task. In its original form, the MET comprises both a 52-item melodic subtest and a 52-item rhythmic subtest. For the purposes of time efficiency, we used only the first 20 items of the melodic subtest, so as to shorten its length to approximately 4 minutes. The Spearman–Brown prophecy formula^63^ indicated that the shortened test should still have good internal reliability (estimated Cronbach’s α = 0.90).

*Adaptive temporal order test:* The *adaptive temporal order test* comprised a computerised adaptive version of the psychoacoustic *Temporal order for tones test* of Kidd and colleagues^64^. It was constructed for the present study using an IRT formulation of the adaptive maximum-likelihood procedure^35^, and using item parameters estimated from the figures provided in the original paper^64^.

The test used a 3-AFC procedure. The task was to discriminate the order of two tones of equal duration, one of 550 Hz frequency, the other of 710 Hz frequency. Item difficulty was determined by manipulating the length of these two tones. The two tones were played without a gap between them, and were preceded and followed by 100-ms ‘leader’ and ‘trailer’ tones of 625 Hz frequency.

The test administered 20 items from an item bank of 800 items, with the length of the target tones permitted to vary between 20 ms and 200 ms. It was implemented using the R package catR^27^ on the Concerto platform^54^.

*Musical training questionnaire:* This study used the same Gold-MSI musical training questionnaire as Study 1.

#### Procedure

*Nationally representative group:* Participants from the nationally representative group took the test in two waves. All participants from the group participated in Wave 1, and 42 of these participants additionally participated in Wave 2. Participants were emailed approximately a week after participation in Wave 1 and given the opportunity to participate in Wave 2 in exchange for additional financial remuneration.

Wave 1 comprised an online test battery of approximately 30 minutes in length and administered using the Concerto platform^54^. The test battery started with the adaptive melodic discrimination test. Apart from the item selection algorithm, the test’s implementation was identical to the implementation in Study 1, except for the fact that participants did not receive any feedback about their final score. Participants then took a prototype version of another musical listening test (results not reported here), followed by the Gold-MSI musical training questionnaire and a short demographic questionnaire asking about the participant’s age, gender, and occupational status. The test battery concluded with the 20-item version of the MET’s melodic subtest.

Participants took part in Wave 2 approximately one week after participating in Wave 1. Wave 2 comprised an online battery of approximately 20 minutes in length, also administered using the Concerto platform. The test battery comprised the adaptive melodic discrimination test, the other prototype test, and the adaptive temporal order test. All details of the implementation of these tests were identical to Wave 1.

*Self-selected group:* Participants from the self-selected group took one online test battery approximately 20 minutes in length, administered using the Concerto platform. The test battery began with the adaptive melodic discrimination test, continued with the other prototype musical test (results not reported here), and concluded with the same musical training and demographic questionnaires administered to the nationally representative group. At the end of the test battery participants were given feedback about the scores that they had achieved in the tests.

### Results

#### Person fit

Participant response patterns were screened for poor fit using Snijder’s^65^ *l*_*z*_ * statistic. No participants exhibited statistically significant poor fit (one-tailed test, *p* > 0.05), and so all participants’ data were retained for further analysis.

#### Population norms

Distributions of melodic discrimination abilities are plotted in Fig. 3. Melodic discrimination abilities in the nationally representative group (*M* = −1.19, *SD* = 1.08) were significantly lower than in the self-selected group (*M* = 0.22, *SD* = 0.96), according to a Wilcoxon rank sum test with continuity correction (*W* = 1624, *p* < 0.001). Likewise, musical training scores in the nationally representative group (*M* = 16.4, *SD* = 9.3) were significantly lower than in the self-selected group (*M* = 28.9, *SD* = 8.9; *W* = 1481.5, *p* < 0.001).

#### Nomothetic span

The nomothetic span of the adaptive melodic discrimination test was assessed in terms of concurrent validity (correlations with MET scores), convergent validity (correlations with musical training scores), and divergent validity (correlations with adaptive temporal order test scores). Correlations were computed using all participants who took the pairs of relevant tests (Table 3); since different sample groups took different tests, the correlations are based on different sample groups and have different degrees of freedom. Two sets of melodic discrimination scores were available for correlating with temporal order test scores, but we solely used the melodic discrimination scores collected in the same testing session as the temporal order test (Wave 2). All three validity types were supported by the results: ability scores were significantly correlated with the concurrent and convergent validity measures, but were not significantly correlated with the divergent validity measure.

**Table 3:** Results of nomothetic span comparisons for test Version 1.

Comparison	Type	Sample group	Pearson correlation (20 items)		
			*r*	*df*	*p*
MET	Concurrent	Nationally representative (Wave 1)	0.530	183	<0.001
Musical training	Convergent	All participants	0.437	236	<0.001
Temporal order	Divergent	Nationally representative (Wave 2)	0.244	40	0.119

**Figure 4:**
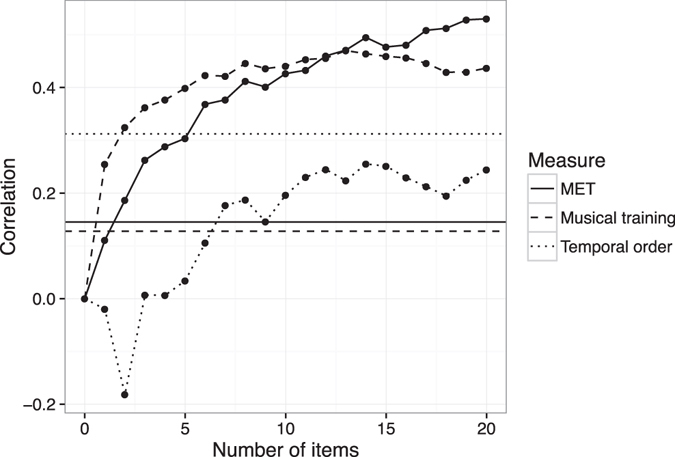
Pearson correlations with validity measures as a function of test length (test Version 1). The three horizontal lines indicate two-tailed thresholds of statistical significance at *p* < 0.05 for the respective validity measures.

Nomothetic span was then examined as a function of test length (Fig. 4). Correlations with musical training scores rose quickly and plateaued early at about 10 items (*r* = 0.44). Correlations with the MET rose slower but for longer, with a moderate correlation (*r* = 0.40) at a test length of 10 increasing to a more substantial correlation (*r* = 0.53) at a lest length of 20. Lastly, correlations with the temporal order test plateaued at a statistically insignificant correlation of *r* = 0.24 after about 12 items.

#### Reliability

**Figure 5:**
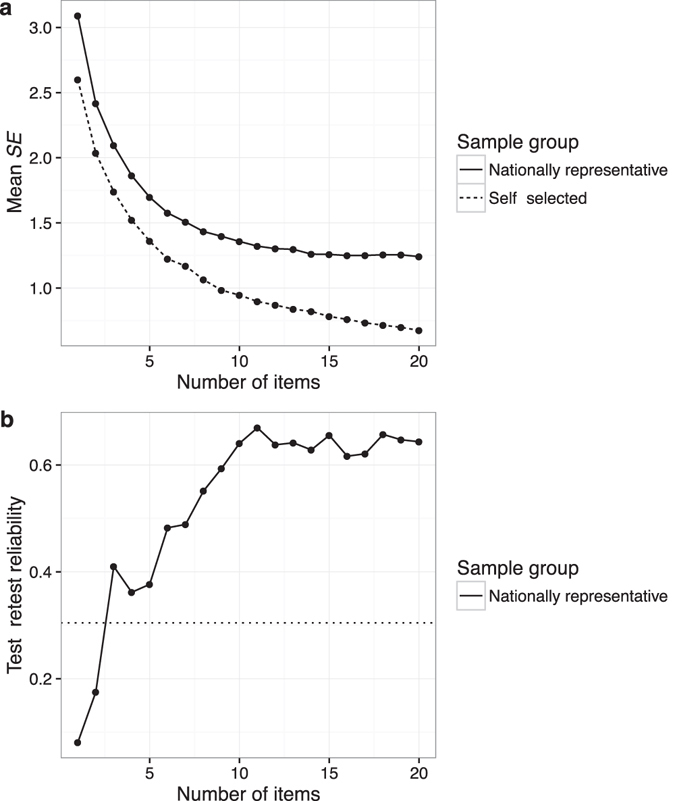
Reliability as a function of test length and sample group (test Version 1), as assessed by (**a**) mean *SE* of ability estimates and (**b**) test-retest reliability (Pearson correlation). The dotted line in (**b**) indicates the threshold of statistical significance at *p* < 0.05 (two-tailed).

Test reliability was first examined using the standard error (*SE*) of the ability estimates as estimated by the IRT model (Fig. 5a). Low *SE* corresponds to high reliability. For the nationally representative group, mean *SE* plateaued at around 15 items with a value of 1.24. Mean *SE* was universally lower for the self-selected group, and continually decreased as test length increased, reaching a value of 0.68 after 20 items. The difference in *SE* between sample groups was statistically significant at *p* < 0.05 for all test lengths except for a test length of three, which had *p* = 0.079 (Wilcoxon rank sum tests with continuity correction).

Test-retest reliability was then examined by computing Pearson correlations between ability estimates for the nationally representative participants that took the test twice (Fig. 5b). Reliability rose quickly to a value of *r* = 0.67 with 11 items, and plateaued for longer test lengths. Intraclass correlations showed a similar pattern.

### Discussion

The aim of this study was to validate Version 1 of the adaptive melodic discrimination test, investigating ability norms, nomothetic span, and test reliability.

Ability norms were significantly lower for the nationally representative group than for the self-selected group. This seems reasonable, as people who actively volunteer for music psychology studies are likely to possess a higher-than-average interest in music, and are likely to be better motivated for the study.

Nomothetic span was supported by measures of concurrent validity, convergent validity, and divergent validity. High correlations with MET scores indicated good concurrent validity, as the MET has been shown to be a reliable measure of musical expertise^4^. High correlations with musical training scores indicated good convergent validity, since previous studies have found melodic discrimination ability to be associated with musical experience and training^4, 6, 11^. Lastly, low correlations with temporal order discrimination scores indicated good divergent validity, as temporal order discrimination is a low-level psychoacoustic task that is ostensibly unrelated to melody discrimination. These three validity measures were robust to test shortening, with good validity being observed for test lengths of about 10 items.

Two reliability measures were computed: one IRT-based measure (ability *SE*s derived from the IRT model) and one empirical measure (test-retest reliability). The former has the advantage that it does not require participants to take the test twice, whereas the latter has the advantage that it does not rely on any IRT assumptions. For both measures, test reliability plateaued for the nationally representative group at about 10–15 items. According to the *SE* measure, however, test reliability was substantially better (lower *SE*) for the self-recruited group, and did not produce any plateau with test length. This discrepancy was likely due to the differing ability levels of the two groups: the item bank did not contain easy enough items for the nationally representative group, resulting in decreased test reliability as compared to the self-recruited group.

In conclusion, Version 1 of the melodic discrimination test showed promise in terms of reliability and validity, but the validation highlighted the need for the item bank to include additional easy items for administration to lower-ability participants. This motivated the construction of the second version of the test.

### Study 3: Second calibration

Version 2 of the melodic discrimination test was designed to address the lack of easy items in Version 1 of the test. New items were constructed by modifying the AIG process to produce additional shorter melodies (see ‘Test Design’ for details). Based on the results of Study 1, it was predicted that these shorter melodies should result in lower item difficulties. The purpose of this study was then to estimate psychometric parameters for these new items, so that the items could be used as part of the adaptive test.

### Method

#### Participants

Two participant groups were used: one self-selected group (*N* = 110) and one group recruited from the crowdsourced music testing company ‘Slicethepie’ (*N* = 118; www.slicethepie.com; the switch to this company from Qualtrics was prompted by financial considerations). Self-selected participants were recruited similarly to Studies 1 and 2, and were approximately evenly split by gender (52 males, 58 females). These participants ranged in age from 18 to 74 years (*M* = 31.7, *SD* = 13.9), and most were either at university (49 out of 110) or in full-time employment (38 out of 110). Meanwhile, the music-testing group comprised more females than males (78 vs. 38), and ranged in age from 15 to 67 years (*M* = 30.1, *SD* = 10.8). These participants represented a greater range of occupational backgrounds (38 in full-time employment, 20 at university, 16 self-employed, 16 unemployed, etc.). No participants in either group reported hearing problems.

#### Materials

*Melodic discrimination test:* This study used a non-adaptive instance of Version 2 of the melodic discrimination test. Version 2 contained 12 new item families not found in Version 1; this study used items from each of these 12 item families, as well as the 8 item families from Version 1 corresponding to the two lowest pre-existing length levels (6 and 7 notes). Items were generated in the same manner as in Study 1, by factorially combining base melodies with tonality violation, contour violation, and position of the odd-one-out. 720 new items were created for the new item families, whereas items for the old item families were reused from Studies 1 and 2.

#### Procedure

Data were collected using the *Concerto* platform^54^. Participants took part online, agreeing to wear headphones and to take the test in a quiet room free from interruptions. Testing sessions began with the non-adaptive melodic discrimination test. As in Study 1, participants first took part in a training phase, and then were administered 20 randomly-selected items, with the constraints that each item family was represented exactly once and that no base melody was heard more than once. After completing the melodic discrimination test, participants then answered a short questionnaire about basic demographic details. At the end of their testing session, self-selected participants were given their melodic discrimination score.

### Results

#### Item parameter estimation

Response data were modelled analogously to Study 1, using the item response model described in equation (2) with *n* = 3. Item family fit was assessed using Yen’s^57^ *Q*_1_ statistic with 10 ability groups, and calculating significance levels using 500 Monte Carlo samples and Bonferroni correction. No cases of significantly poor fit were found, a result that proved robust to variation of the number of ability groups. Model fit and conditional independence were assessed by inspecting the two- and three-way margins^58^: none of the two-way pairs were flagged, and only 49 out of 9,120 (0.54%) three-way pairs were flagged by Bartholomew’s^58^ criterion (test statistic greater than 4.0). Modified parallel analysis^59^ found no evidence for multidimensionality (500 Monte Carlo samples, *p* = 0.95). In summary, these results indicated that the IRT model satisfied the important assumptions of model fit, conditional independence, and unidimensionality.

The newly estimated item parameters were then mapped onto the metric of the previously estimated item parameters using mean-mean equating^66^ as implemented in the *equateIRT* package^67^ in R^56^. This involves computing equating coefficients *A* and *B*, which define a mapping from old discrimination (*a*) and difficulty (*b*_*j*_) parameters to new discrimination and difficulty parameters $(a^{\prime} ,{b}_{j}^{^{\prime} })$ as follows^67^:

$$a^{\prime} =\frac{a}{A}$$

$${b}_{j}\text{'}=A{b}_{j}+B$$

The optimised values of *A* and *B* were *A* = 1.27 (*SE* = 0.13) and *B* = 0.01 (*SE* = 0.13). The small size of the standard errors suggested that the linking process was relatively successful, and therefore could be used to incorporate the new item families into the test’s item bank.

#### Ability distributions

Melodic discrimination ability scores (expected *a posteriori*) were calculated for all participants (Fig. 3). The mean estimated ability in the self-selected group was 0.45 (*SD* = 0.87), whereas the mean estimated ability in the music testing group was −0.42 (*SD* = 0.66). The difference in abilities between the two groups was statistically significant as assessed by a Wilcoxon rank sum test with continuity correction (*W* = 10202, *p* < 0.001).

#### Comparing item parameters to ability parameters

Figure 3 shows that test Version 2 has better coverage at lower difficulty ranges than test Version 1, while maintaining coverage at higher difficulty ranges. The resulting item difficulty distribution provides good coverage for the two sample groups tested in Study 3 (crowdsourced music testers and self-selected adults). However, it still does not contain sufficiently easy items for the lowest-ability participants observed in Studies 1 and 2, particularly in the case of the nationally representative adults and the schoolchildren.

### Discussion

The purpose of this study was to calibrate Version 2 of the melodic discrimination test. Version 2 was intended to address the lack of easy items in Version 1, and hence improve the test’s discrimination performance for low-ability participants. New easy items were created by running the automatic item generation process with a specification for shorter melodies, on the basis of the relationship between melody length and item difficulty observed in Study 1. These new items were then calibrated empirically, and their parameters were linked with the parameters of previously calibrated items to produce a coherent item bank.

The results indicated good IRT model fit for the new items, supporting their use in the new melodic discrimination test. They also indicated that the new items provide extra coverage at the low end of the difficulty spectrum, suggesting that the test should now perform better for low-ability participants.

It was surprising that some participants in Studies 1 and 2 still fell below the ability range covered by the newly calibrated test items. We originally hypothesised that reducing melody length to three notes should make the memory task trivial, with contour violations being particularly easy to detect. Perhaps some participants performed especially poorly on account of clinical impairments (e.g. amusia^21^). Others may have performed poorly due to inattention, task misunderstanding, or defective listening equipment. Alternatively, the 3-AFC task might simply place too high demands on auditory working memory for some participants, irrespective of melody length. It would be worth exploring these potential explanations in a future laboratory study. Nonetheless, the new test can still make ability estimates at these low ability levels, just with lower precision.

### Study 4: Second validation

The aim of this final study was to investigate the reliability of Version 2 of the adaptive melodic discrimination test. This was addressed using a combination of empirical data collection and data simulation.

### Method

#### Participants

The participant group was recruited through the same music testing company used in Study 3. There were 162 participants in total, with slightly more than half being female (91 females, 69 males, two declining to report gender). Stratified sampling was used to achieve an age distribution approximately representative of the adult UK population, and participants ranged in age from 17 to 75 years (*M* = 36.8, *SD* = 14.0). Most participants were either in full-time employment (60 individuals), self-employed (36 individuals), or at university (24 individuals). No participants reported hearing problems.

#### Materials

*Melodic discrimination test:* This study used Version 2 of the adaptive melodic discrimination test, using item parameters estimated in Studies 1 and 3. The same adaptive procedure was used as in Study 2, and the item family selected for the starting item remained the same as in Study 2. The online infrastructure remained the same as in previous studies.

#### Procedure

Empirical data were collected using the same procedure as in Study 3, except for the fact that participants were given an adaptive test rather than a non-adaptive test, and that no participants were given feedback about their performance.

In addition, a simulation experiment was conducted to compare the performance of different test versions. The *catR* package^27^ was used to simulate item response patterns in various versions of the melodic discrimination test. Each simulation used 10,000 simulated participants drawn from a normal distribution matched to the observed ability distribution in the empirical part of this study. Simulations were run for every test length between one and 20 items, and for three different melodic discrimination tests:

- Version 1, with randomised (i.e. non-adaptive) item selection;
- Version 1, with adaptive item selection;
- Version 2, with adaptive item selection.

### Results

#### Person fit

Participant response patterns were screened for poor fit using Snijder’s^65^ *l*_*z*_ * statistic. No participants exhibited statistically significant poor fit (one-tailed test, *p* > 0.05), and so all participants’ data were retained for further analysis.

#### Ability distribution

The mean observed ability in the empirical data was −0.42 (*SD* = 1.16). This mean ability was approximately halfway between the mean abilities observed for the two sample groups in Study 2 (nationally representative group, *M* = −1.19; self-selected group, *M* = 0.22; Fig. 3).

#### Standard error of ability estimates

**Figure 6:**
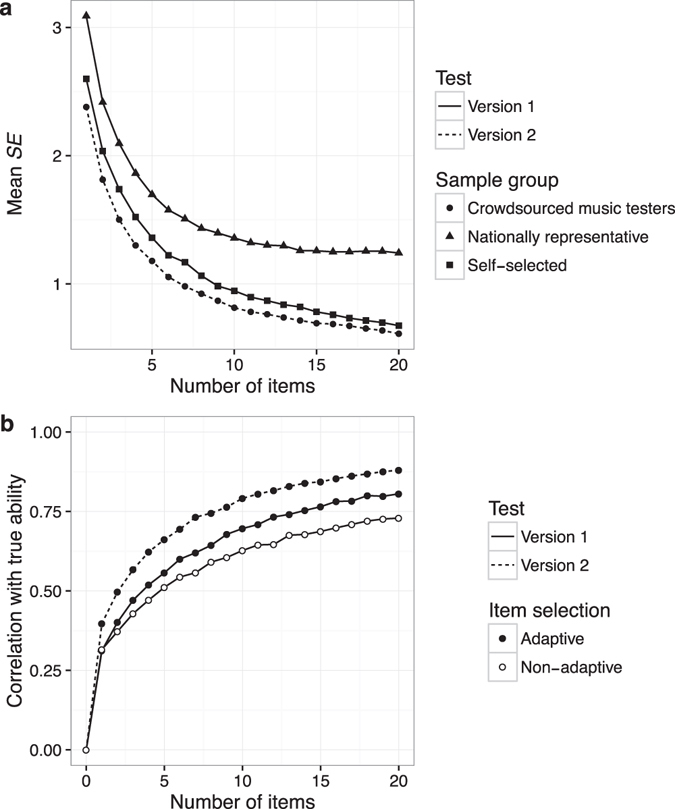
Reliability as a function of test length, sample group, and test version, as assessed by (**a**) mean *SE* of ability estimates and (**b**) Pearson correlation between estimated abilities and true abilities (simulation experiment).

Mean estimated standard errors (*SE)* of ability estimates were compared with results obtained for the previous test version (Fig. 6a). Version 2 of the test obtained better reliability than Version 1 for all sample groups tested (pairwise Wilcoxon rank sum tests with continuity correction, *p* < 0.05 at all time points) with a 10-item mean *SE* of 0.81 and a 20-item mean *SE* of 0.61.

#### Simulated reliability

A second measure of reliability was calculated by simulating the Pearson correlation between ability estimates and true ability scores (Fig. 6b). Version 2 of the adaptive melodic discrimination test outperformed the adaptive Version 1 test, achieving a correlation of *r* = 0.79 with 10 items and *r* = 0.88 with 20 items. The adaptive Version 1 test, meanwhile, outperformed the non-adaptive Version 1 test, with a correlation of *r* = 0.70 (vs. *r* = 0.63) for 10 items and *r* = 0.81 (vs. *r* = 0.73) for 20 items. These differences in correlations were statistically significant for all test lengths greater than zero, according to Fisher’s^68^ *z* test (*p* < 0.05) as computed by the *cocor* package^69^, except when the test contained only one item, in which case adaptive and non-adaptive implementations of test Version 1 were not significantly different (*p* = 0.786).

### Conclusion

This study investigated the reliability of Version 2 of the adaptive melodic discrimination test. Both empirical data and simulation data suggested that Version 2 provides substantial performance improvements upon Version 1, likely due to achieving better coverage at the low end of the ability spectrum. Additionally, the simulation study indicated that both adaptive tests substantially outperform the non-adaptive Version 1 test, suggesting therefore that adaptive testing makes an important contribution to the test’s reliability.

## General Discussion

The aim of this paper was to demonstrate the application of several modern psychometric techniques to the construction of a melodic discrimination test. These techniques included item response theory (IRT), computerised adaptive testing (CAT), and automatic item generation (AIG). Such techniques have been explored in detail in the field of psychometrics, but have yet to achieve mainstream usage in psychological research, especially in music psychology. This is unfortunate, as together these techniques can bring a number of advantages over traditional testing methods, including flexible test lengths, sophisticated reliability measures, improved testing efficiency, improved construct validity, avoidance of exposure effects, and improved test-construction efficiency.

The four studies conducted here support the viability of the new melodic discrimination test. Construct validity was assessed both in terms of construct representation and nomothetic span. Study 1 provided strong support for the test’s construct representation by successfully modelling the relationship between structural item features and item difficulty. Studies 1 and 2 supported the test’s nomothetic span by analysing relationships between test scores and other person-level variables. Studies 1 and 3 showed that the test satisfies the standard assumptions of IRT modelling. Studies 2 and 4 showed evidence for the test’s reliability, both in terms of empirical test-retest correlations, IRT-based standard error estimates, and simulated reliability.

This work has therefore resulted in the construction of a well-validated and psychometrically sophisticated melodic discrimination test. This test should be useful for subsequent music psychology research, providing a way to assess a core aspect of musical ability efficiently and objectively. The test could also have useful educational applications, such as helping to identify appropriate streams for music classes, or to assess listening abilities as part of music scholarship exams.

Melodic discrimination is, however, just one of many aspects of musical sophistication. Effective all-round musical testing will necessitate extending these modern psychometric techniques to a range of different musical abilities, such as the ability to identify intended emotions in expressive music performance^70^, to discriminate rhythmic patterns^4–6^, and to entrain to a beat^37, 38^. Some of these abilities will be more challenging to assess than others; in particular, tasks that contain subjective aspects such as emotion inference might prove to be harder to operationalise than objective tasks such as melody discrimination. Nonetheless, the present paper should provide a useful framework for addressing such problems.

This paper has focused primarily on the musical domain, but the techniques described here should generalise well to other areas of individual differences research, such as emotional intelligence^71^ or facial processing^72^. Different applications present different challenges: an emotional intelligence test might require algorithmic generation of text describing emotional scenarios, whereas a facial processing test might require algorithmic generation of pictures of human faces. However, the underlying psychometric principles remain constant, as do the potential rewards such as improved construct validity and test efficiency.

The present research was primarily geared towards online testing. Much of the test calibration data were collected online, as were all of the test validation data. We suggest that our new melodic discrimination test should also be suitable for laboratory use, but some differences between the two testing environments should be borne in mind. Generally speaking, laboratory testing environments are likely to facilitate melodic discrimination compared to online testing environments, since psychology laboratories typically have minimal background noise and distractions. This is likely to result in higher estimated participant abilities. Moreover, laboratory testing environments are likely to be more consistent than online testing environments, since laboratory participants all experience the same environment, and this environment does not change during the test. This is likely to improve the test’s reliability.

The accuracy of item difficulty predictions has a direct impact on test reliability. Our results suggest that our item difficulty predictions are fairly accurate, but a more accurate predictive model would always be possible. This could be achieved in several ways, perhaps by using a larger calibration sample, controlling for a greater number of structural item features, or using a more sophisticated IRT model. We aim to explore these possibilities in future work.

Most IRT models assume that performance levels stay constant throughout a test. However, real-life situations often violate this assumption. For example, as the test progresses, performance might be aided by learning effects, or alternatively impaired by fatigue effects. In the present work we aimed to minimise such confounds by including practice trials and keeping test lengths short, but some item-position effects may have remained nonetheless. One interesting possibility for future work would be to address these item-position effects by incorporating them explicitly into the psychometric model (e.g. ref. 73).

Given that the main benefit of CAT is improved testing efficiency, it would be interesting to compare the reliability of our test with that of pre-existing melodic discrimination tests. Unfortunately the reliability coefficients reported by previous studies are all sample-dependent, making it difficult to make meaningful comparisons between tests. A proper comparison of reliability would entail administering multiple tests to the same participant group and computing test-retest correlations. This would be a time-consuming endeavour, but would produce a useful comparison between the various tests available.

The melodic discrimination test described in this paper was constructed specifically with Western listeners in mind. Two aspects of the test are particularly Western-centric: the use of Irish folk melodies and the manipulation of tonality. Irish folk melodies share many stylistic aspects with Western music as a whole, and hence conform to musical schemata with which most Western listeners are familiar. However, non-Western listeners may well be less familiar with these schemata, resulting in impaired melodic discrimination performance and lower test scores^74^. Likewise, the salience of tonality violations to Western listeners likely derives from an implicit knowledge of Western tonal structure learned on the basis of passive exposure to Western tonal music^75^. Tonality violations may therefore be less salient to non-Western listeners who have not received the same exposure to Western tonal music, impairing melodic discrimination performance and lowering test scores as a result. These cultural biases should be taken into account when administering the new melodic discrimination test to non-Western listeners. An interesting way to address these biases might be to develop different test versions for different musical cultures, perhaps by training the melody generation model on non-Western corpora instead of Irish folk music, and recalibrating the psychometric model on response data from non-Western listeners.

The current version of the adaptive melodic discrimination test is available upon request from the researchers. We have developed an online implementation using the Concerto platform^54^ which can be used either for online testing or for laboratory testing. Test length can be specified by the researcher according to practical constraints; we suggest a short test (e.g. 10–20 items, 3–6 minutes) when the test is administered together with other individual differences tests, and a longer test (e.g. 20 to 35 items, 6–9 minutes) if the test is administered independently. We anticipate it being a useful tool for subsequent research.

## Electronic supplementary material

Supplementary figures
