# Supplementary material for: Applying modern psychometric techniques to melodic discrimination testing: Item response theory, computerised adaptive testing, and automatic item generation
Source: Sci Rep. 2017 Jun 15;7:3618. doi: 10.1038/s41598-017-03586-z (PMC5472621; doi:10.1038/s41598-017-03586-z)
Supplement: Supplementary file 1 — Supplementary figures [file 41598_2017_3586_MOESM1_ESM.pdf]

Applying modern psychometric techniques to melodic discrimination testing: Item response theory, computerised adaptive testing, and automatic item generation

Supplementary figures

Peter M. C. Harrison[1,2,*], Tom Collins[3,4], and Daniel Müllensiefen[2]

[1]Queen Mary University of London, School of Electronic Engineering and Computer Science, London, E1 4NS, England

[2]Goldsmiths, University of London, Department of Psychology, London, SE14 6NW, England

[3]Lehigh University, Department of Psychology, Bethlehem, PA 18015, USA

[4]Music Artificial Intelligence Algorithms, Inc., Davis, CA 95617, USA

*p.m.c.harrison@qmul.ac.uk

Example items

This section contains four example items from the item bank for Version 2 of the melodic discrimination test. The item families are chosen to illustrate the range of melody lengths available, as well as illustrating contour violations and tonality violations.

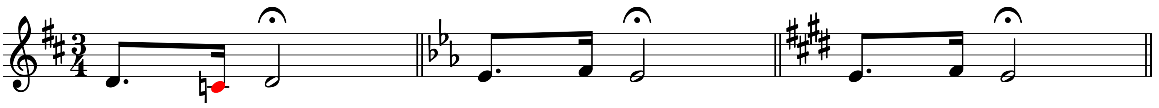

Supplementary Figure S1. *Example item with a length of three notes, contour violated, and tonality violated. The first melody is the odd-one-out, and the altered note is highlighted. This item has an estimated difficulty of −0.991.*

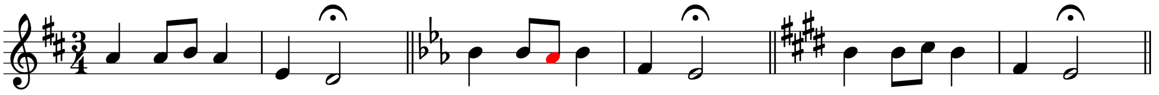

Supplementary Figure S2. *Example item with a length of six notes, contour violated, and tonality preserved. The second melody is the odd-one-out, and the altered note is highlighted. This item has an estimated difficulty of −0.197.*

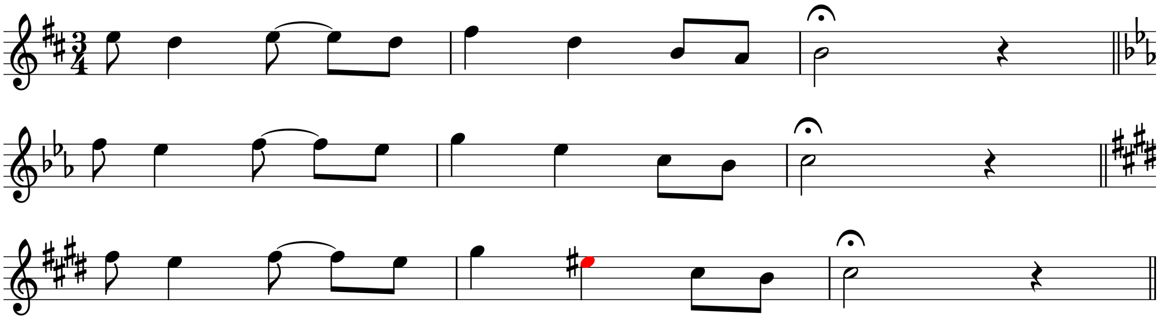

Supplementary Figure S3. *Example item with a length of nine notes, contour preserved, and tonality violated. The third melody is the odd-one-out, and the altered note is highlighted. This item has an estimated difficulty of 0.613.*

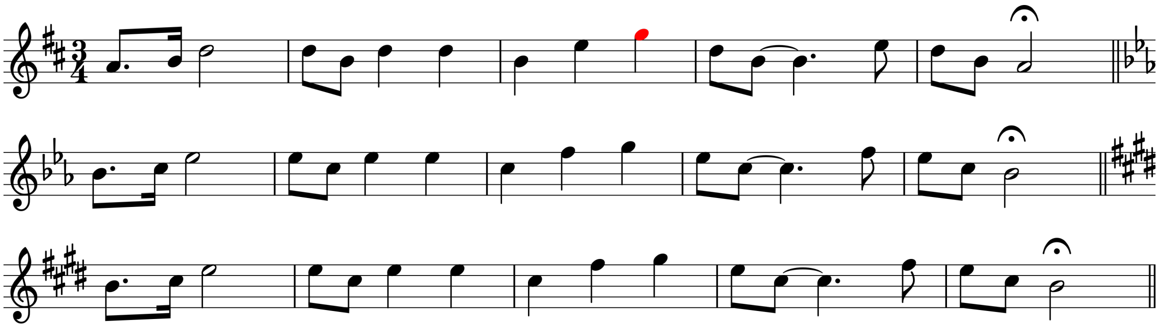

Supplementary Figure S4. *Example item with a length of sixteen notes, contour preserved, and tonality preserved. The first melody is the odd-one-out, and the altered note is highlighted. This item has an estimated difficulty of 1.844.*
